# Assessing adults' beliefs about the motivations for indoor tanning

**DOI:** 10.1111/phpp.12797

**Published:** 2022-05-12

**Authors:** Diem‐Phuong D. Dao, Arsh N. Patel, Caroline L. Porter, Matthew C. Johnson, Thoai T. Vu, Vinh D. Dao, Sara Kakatkar, Steven R. Feldman

**Affiliations:** ^1^ Center for Dermatology Research, Department of Dermatology Wake Forest School of Medicine Winston‐Salem North Carolina USA; ^2^ Department of Pathology Wake Forest School of Medicine Winston‐Salem North Carolina USA; ^3^ Department of Social Sciences & Health Policy Wake Forest School of Medicine Winston‐Salem North Carolina USA; ^4^ Department of Dermatology University of Southern Denmark Odense Denmark

To the Editor,

Indoor tanning through the use of ultraviolet (UV)‐emitting beds is estimated to cause over 450,000 nonmelanoma skin cancers and over 10,000 melanomas in the United States, Europe, and Australia annually.^1^ Despite these health concerns, indoor tanning *still occurs* among U.S. adolescents and young adults. One motivation for tanning may be the desire to improve appearance.^2^ Although indoor tanning rates have decreased in recent years, a subgroup of individuals continue to tan frequently and may exhibit behaviors consistent with addiction.^2^, ^3^ Frequent tanners cite a major reason for their tanning is the relaxing effects of UV light; when given a blinded choice, they choose UV over non‐UV‐emitting tanning beds.^3^ The release of endorphins following UV exposure plays a role in tanning bed dependency, and frequent tanners may experience withdrawal‐like symptoms when exposed to the opioid antagonist, naltrexone.^3^

We assessed beliefs about the motivations behind indoor tanning bed use and aimed to determine if the potentially addictive effects of frequent tanning are recognized by the general adult public.

We surveyed 289 U.S. participants through Amazon Mechanical Turk (MTurk) about their basic demographic characteristics (sex, age, race, ethnicity, highest educational attainment), beliefs about the motivations for indoor tanning, if they agreed that frequent tanners may be addicted to the relaxing effects of endorphins, and their level of surprise about the potentially addictive effects of tanning bed use. The questions were not pretested. Free‐text answers on motivations for indoor tanning were categorized as aesthetic‐based, convenience‐based, both aesthetic‐ and convenience‐based, health‐ or safety‐based, and other. None of the free‐text answers listed “addiction” as a reason for tanning, however, one individual listed “obsession.” This answer was grouped into the “other category.” Aesthetic‐based motivations were grouped based on beliefs that appearances played a major role in tanning motivation. Convenience‐based motivation answers were grouped based on indoor tanning location, timing, and ease of scheduling compared to outdoor tanning. Convenience‐based motivations included responses such as “convenience” or “quick.” Health and safety motivations were grouped based on the perception that indoor tanning either imparted health benefits or provided a safer form of tanning versus outdoor tanning. Participants were also asked about their personal indoor tanning history, such as current or past tanning.

Statistical analyses using SPSS 28 included descriptive statistics and chi‐square test of independence. A chi‐square test of independence was used to determine if there was a difference between tanners and nontanners in awareness of the relaxing effects of tanning. Individuals who responded “I already knew that” or “not surprised” were considered aware, while those who answered “somewhat surprised” or “very surprised” were considered not aware. *P*‐values <.05 were considered to be statistically significant.

All 289 individuals completed the survey, of whom 164 (56.7%) were female, 70 (24.2%) in the age group 18–29 years old, 108 (37.4%) were 30–39 years old, and 109 (37.7%) were 40–80 years old. Caucasian was the dominant race in this study (239 individuals, 82.7%), while “not Hispanic or Latino” was the dominant ethnicity (267 individuals, 92.4%).

A total of 138 (47.8%) participants believed aesthetic motivations are the primary reason for indoor tanning bed use, followed by convenience‐based motivations by 95 (32.9%) individuals (Figure 1). Of the 289 participants, 174 (60.2%) were past or current tanning bed users. Additionally, 153 (52.9%) participants reported they believed frequent tanners may be addicted to the relaxing effects of tanning, while 157 (54.3%) participants agreed frequent tanners may be addicted to the relaxing effect of endorphins.

**FIGURE 1:**
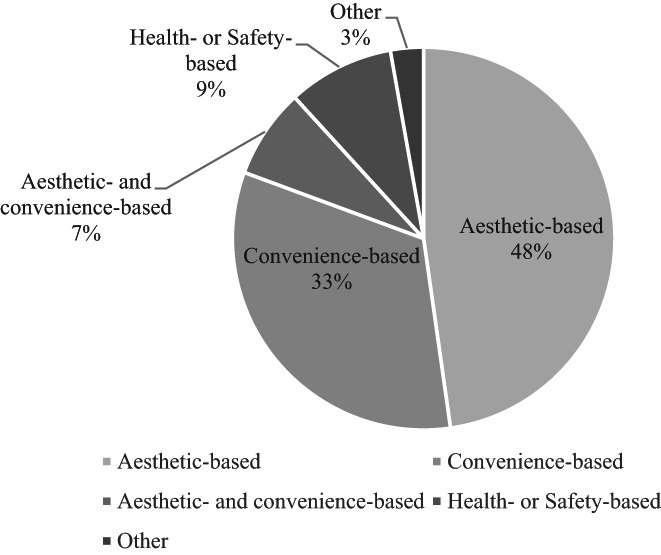
Survey participants' beliefs on the motivation for indoor tanning (*N* = 289)

Few participants (31, 10.7%) reported already knowing UV light exposure from indoor tanning beds caused release of endorphins, while 122 (42.2%) were not surprised (Table 1). Even fewer participants (24, 8.3%) knew frequent tanners may be addicted to the relaxing effect of these endorphins; 147 (50.9%) individuals reported not being surprised (Table 1). Current and past tanners (37.0%) were more likely than nontanners (15.9%) to be aware of the endorphin‐mediated relaxing effects of tanning (χ^2^(1) = 12.84, *p* < .001).

**TABLE 1:** Survey participants' knowledge on the potentially addictive effects of frequent tanning (*N* = 289)

	I already knew that *n* (%)	Not surprised*n* (%)	Somewhat surprised *n* (%)	Very surprised *n* (%)
Ultraviolet light exposure from indoor tanning beds causes release of endorphins	31 (10.7)	122 (42.2)	106 (36.7)	30 (10.4)
Frequent tanners may be addicted to the relaxing effects of these endorphins	24 (8.3)	147 (50.9)	98 (33.9)	20 (6.9)

As previously observed, the main belief on motivations for UV tanning was appearance enhancement.^2^, ^4^, ^5^ Many individuals, including current tanners, are not aware of the endorphin‐mediated, addictive effects of UV tanning. Educating individuals may help curb initial tanning use, but may not be enough to prevent tanning bed use and possible addiction. Although individuals who currently tan or have tanned in the past were more likely aware than nontanners of the association between the relaxing effects of tanning and release of endorphins, tanners still had low rates of awareness. As a result, solely educating individuals on the harmful consequences of tanning may not fully deter them from tanning. Additional interventions are likely needed to reduce the prevalence of indoor tanning and mitigate possible addiction.

A study limitation is we did not assess duration of tanning sessions or length of tanning history. Past or present tanners were not directly asked if they believed they were addicted to tanning or if they believed addiction was a driver for their tanning. As a result, we were not able to assess if past or present tanners were aware of their possible addiction. Another limitation included the possibility of selection bias. However, any participant meeting the inclusion criteria was able to complete the survey regardless of past tanning history or tanning knowledge. Although free‐text motivations categorizations were performed by the research team, categorizations were based on keywords used in or related to the free response answers.

Although aesthetic‐based motivations were the main drivers of indoor UV tanning, a subgroup of individuals reported knowing tanning's endorphin‐mediated relaxing effects may contribute to tanning addiction. The beliefs and underlying motivations fueling tanners with underlying addiction or addictive tendencies may be of relevance as indoor tanning continues despite regulations and public awareness campaigns of the dangers.^6^ Filling these knowledge gaps may allow clinicians to better educate and provide mental health resources, where appropriate, for high‐risk, frequent tanners to circumvent future tanning bed use.

## AUTHOR CONTRIBUTIONS

All authors have made substantial contributions to conception and design, acquisition of data, and analysis and interpretation of data; have been involved in drafting the manuscript and revising it critically for important intellectual content; have given final approval of the version to be published; and agree to be accountable for all aspects of the work in ensuring that questions related to the accuracy or integrity of any part of the work are appropriately investigated and resolved.

## CONFLICT OF INTEREST

Dr. Steven Feldman has received research, speaking, and/or consulting support from Eli Lilly and Company, GlaxoSmithKline/Stiefel, AbbVie, Janssen, Alovtech, vTv Therapeutics, Bristol‐Myers Squibb, Samsung, Pfizer, Boehringer Ingelheim, Amgen, Dermavant, Arcutis, Novartis, Novan, UCB, Helsinn, Sun Pharma, Almirall, Galderma, Leo Pharma, Mylan, Celgene, Ortho Dermatology, Menlo, Merck & Co, Qurient, Forte, Arena, Biocon, Accordant, Argenx, Sanofi, Regeneron, the National Biological Corporation, Caremark, Teladoc, Eurofins, Informa, UpToDate, and the National Psoriasis Foundation. He is the founder and part owner of Causa Research and holds stock in Sensal Health. Ms. Dao, Mr. Patel, Dr. Porter, Dr. Johnson, Mr. Vu, Mr. Dao, and Ms. Kakatkar have no conflicts to disclose.

## Data Availability

Research data are not shared.
